# Retraction: Metallic ions encapsulated in electrospun nanofiber for antibacterial and angiogenesis function to promote wound repair

**DOI:** 10.3389/fcell.2022.1058556

**Published:** 2022-10-11

**Authors:** Frontiers Editorial Office

Following publication, concerns were raised regarding the validity of the data in the article. The authors were unable to provide the complete raw dataset or a satisfactory explanation during the investigation, which was conducted in accordance with Frontiers’ policies. Given the concerns and the fact that raw data were only partially provided, the editors no longer have confidence in the findings presented in the article.

This retraction was approved by the Chief Editors of Frontiers in Cell and Developmental Biology and the Editor-in-Chief of Frontiers. The authors did contact the Editorial office spontaneously to rectify the scientific record, and thus agree to the retraction.

